# Genotoxicity of Polycyclic Aromatic Hydrocarbons and Nitro-Derived in Respirable Airborne Particulate Matter Collected from Urban Areas of Rio de Janeiro (Brazil)

**DOI:** 10.1155/2013/765352

**Published:** 2013-05-02

**Authors:** Claudia Ramos de Rainho, Sérgio Machado Corrêa, José Luiz Mazzei, Claudia Alessandra Fortes Aiub, Israel Felzenszwalb

**Affiliations:** ^1^Universidade do Estado do Rio de Janeiro, Instituto de Biologia Roberto Alcantara Gomes, Departamento de Biofísica e Biometria, Laboratório de Mutagênese Ambiental, 20551-030 Rio de Janeiro, Brazil; ^2^Universidade do Estado do Rio de Janeiro, Faculdade de Tecnologia, Departamento de Química Ambiental, 27537-000 Resende, RJ, Brazil; ^3^Fundação Oswaldo Cruz, Farmanguinhos Plataforma de Métodos Analíticos, 21040-360 Rio de Janeiro, Brazil; ^4^Universidade Federal do Estado do Rio de Janeiro, Instituto Biomédico, Departamento de Genética e Biologia Molecular, Laboratório de Genotoxicidade, 20211-040 Rio de Janeiro, Brazil

## Abstract

Air pollution toxic effects are mainly attributed to small inhalable particulates with an aerodynamic diameter of less than 2.5 **µ**m (PM 2.5). Our objective was to investigate mutagenic and clastogenic activity in PM samples collected in Rio de Janeiro. Samples were collected using a high-volume sampler at three sites: with low traffic and (2) and (3) with a heavy traffic. Six polycyclic aromatic hydrocarbons (PAHs) were quantified by gas chromatography/mass spectrometry (GC/MS). *Salmonella typhimurium* TA98 and the derivative strains YG1021 and YG1024 were used in mutagenicity assays in the presence of organic extracts (10–50 **µ**g/ plate) with and without exogenous metabolization. *Allium cepa* test was performed to evaluate possible cytotoxic and clastogenic activities. The highest PM 2.5 **µ**m (132.73 **µ**m/m^3^) and PAH values (1.22 ng/m^3^ for benzo(a)pyrene) were detected at site 3. High mutagenic frameshift responses in absence and presence of metabolic activation were detected at site 3. The participation of nitroarenes and dinitroarenes was detected in the total mutagenicity of the extracts studied. The cytotoxic effect and the abnormalities detected by *Allium cepa* test can be attributed to the PAH nitroderivatives in the organic extracts. Evaluation of the genotoxicity of urban airborne particulate matter is important as a basis for decision making by regulatory authorities.

## 1. Introduction

Epidemiological studies have demonstrated that exposure to urban particulate matter (PM) is associated with several adverse health effects. Long-term exposure to high concentrations of PM increases the risk of lung cancer, respiratory diseases, and arteriosclerosis, whereas short-term exposure peaks can cause exacerbation of several forms of respiratory diseases, including bronchitis and asthma, as well as changes in heart rate variability [1, 2]. Studies conducted in Rio de Janeiro city showed an association between PM exposure and mortality from lung cancer [3, 4], reduced respiratory capacity amongst children [5], and increased pediatric visits to treat symptoms of bronchial obstruction [6].

The toxic effects of PM are mainly attributed to small inhalable particulates with an aerodynamic diameter of less than 2.5 *μ*m (PM 2.5). Because of their large specific surface, these particulates can adsorb various organic substances, such as polycyclic aromatic hydrocarbons (PAHs), nitroaromatic hydrocarbons (nitro-PAHs), and oxygenated aromatic hydrocarbons (oxy-PAHs) [7]. According to [8], the mutagenicity of airborne particulate can result from at least 500 identified compounds from various chemical classes. Mutagenic nonsubstituted PAHs are the most studied and well-known compounds; however, more recently, nitro- and oxy-PAHs have been shown to be very important because of their strong biological activity [9–11].

The *Salmonella*/*microsome* microsuspension assay has been used for large, multisite, and/or time-series studies, for bioassay-directed fractionation studies, for identifying the presence of specific classes of mutagens, and for siteor source-comparisons of relative levels of airborne mutagens [8]. The use of strains of *Salmonella typhimurium* with different metabolic capacities can indicate the class or classes of compounds present in an environmental sample [11–13].

Higher plants are recognized as excellent genetic models to detect environmental mutagens and are frequently used in monitoring studies. Among the plant species, *Allium cepa* has been used to evaluate DNA damages, such as chromosome aberrations and disturbances in the mitotic cycle. Employing the *Allium cepa* as a test system to detect mutagens dates back to the 30s. It has been used to this day to assess a great number of chemical agents, which contributes to its increasing application in environmental monitoring. It is easily handled and has advantages over other short-term tests that require previous preparations of tested samples, as well as the addition of exogenous metabolic system [14].

The objective of the present work was to investigate mutagenic and clastogenic activities in the PM samples collected at three sites in Rio de Janeiro.

## 2. Material and Methods

### 2.1. Sampling Sites

The samples were collected at three sites in Rio de Janeiro: the campus of the Rio de Janeiro State University (site 1), Avenida Brasil (site 2), and Rebouças tunnel (site 3) between August and October 2010 (winter and spring seasons). Site 1, with low traffic, is located in a residential area of the city's north zone. Site 2 has heavy traffic (~250,000 vehicles/day) and is the city's biggest highway, covering 58 km in length and crossing 27 neighborhoods. Site 3 has heavy traffic (~190 000 vehicles/day). It connects the north and south zones of the city and is 2.8 km long [15].

Airborne PM 2.5 samples were collected on fiberglass filters (E558 X 10IN, 254 mm × 203 mm) using a high-volume collector (AVG MP 2.5, 1.13 m^3^/mim) for 24 h for site 1 and site 2 and 6 h for site 3. Four monthly samplings were performed for each site of study. The filters were weighed and stabilized before and after sampling (45% humidity) for the determination of particulate concentration, expressed in *μ*g/m^3^ units of sampled air [16–18]. At the end of the sampling, the filters were combined to form a pool sample. 

### 2.2. Extraction of Organic Compounds

Half of each filter was sonicated in three rounds of 10 min each using dichloromethane (DCM, CASRN. 75-09-2, TediaBrazil, Brazil, purity 99.9%). The extracts were concentrated to 15 mL in a rotating evaporator and filtered in a Teflon membrane (0.5 *μ*m). The concentration of extractable organic matter (EOM, in *μ*g/m^3^) was calculated. Prior to bioassays, the organic extract was dried at 4°C and resuspended in 5 *μ*L dimethyl sulfoxide (DMSO, CASRN. 67-68-5, Synth, Brazil, purity 99.9%) [16–18].

### 2.3. Analysis of Polycyclic Aromatic Hydrocarbons (PAHs)

PAHs were identified and quantified by gas chromatography/mass spectrometry (GC/MS), using a Varian system consisting of a gas chromatograph (450-GC) with a split/splitless injector 1177S/SL (kept at 300°C) coupled to the mass spectrometer detector (MS 220). The ion trap (250°C), manifold (280°C), and transfer line (280°C) were maintained at constant temperatures. PAHs were identified by mass similarity and by the retention time of the components in a commercial standard kit (Supelco, PAH610-S). Quantification was based on five calibration points, which were constructed from each standard for all the target analytes, ranging from 10 to 250 pg/*μ*L. Injections (2.0 *μ*L) were splitless, with the split opened after 0.5 min, and helium 5.0 was used as the carrier gas. A VF-5MS column (30 m × 0.25 mm × 0.25 *μ*m) was employed. The column and septum purge flows were set at 1.6 and 3 mL/min, respectively. The oven temperature program was as follows: 70°C for 4 min then heating to 300°C at 10°C/min. This directive was designed for the analysis of the sixteen main priority PAHs, but only six were detected: phenanthrene, fluoranthene, pyrene, benzo(a)anthracene, chrysene, and benzo(a)pyrene. The limits of quantification were determined from the minimum point in the calibration curves. Limits of detection were determined from PAH concentrations, which resulted in a signal-to-noise ratio of 3 : 1. The results were expressed in ng/m^3^ [19].

### 2.4. *Salmonella*/*Microsome* Assay

The organic extracts were assayed for mutagenicity using the microsuspension version [20] of the *Salmonella/microsome* assay [21]. *Salmonella typhimurium* TA98 (frameshift strain) and the derivative strains YG1021 (nitroreductase overproducing) and YG1024 (*O*-acetyltransferase overproducing) [22] were used, with and without metabolic activation (S9 mix fraction). Five concentrations of each sample (10, 20, 30, 40, and 50 *μ*g/plate) were tested in triplicate. The samples were preincubated for 90 min. All assays were carried out under yellow light and in the presence of negative (dimethyl sulfoxide-DMSO solvent, 5 *μ*L/plate) and positive (4-nitroquinoline oxide-4NQO, 0.5 *μ*g/plate, CASRN. 56-57-5 and 2-aminofluorene-2AF, 1 *μ*g/plate, CASRN. 153-78-6 from Sigma Chemical Company, St. Louis, MO, USA) controls. Plates were incubated in the dark at 37°C for 72 h; after which time, revertants were counted. The sample was considered positive when a mutagenesis value of at least twice the negative value, a significant ANOVA (*P* < 0.05), and a positive dose-response rate (*P* < 0.05) were observed. The results of the different assays were analyzed via the SALANAL program (Salmonella Assay Analysis, version 1.0, Integrated Laboratory Systems of Research Triangle Institute, RTP, North Carolina, USA). The choice between linear regression and the Bernstein model [23] was made to allow the elimination of data for doses outside the linear portion of the dose-response curve. Positive results were interpreted as presenting significant mutagenicity. Positive responses were expressed as the number of revertants per volume of air sampled (rev/m^3^), that is, rev/*μ*g multiplied by EOM in *μ*g/m^3^. In the cytotoxicity test, the solution containing the sample and the bacterial culture (100–200 cells) were plated on nutrient agar plates and incubated at 37°C for 24 h, and the surviving colonies were counted. The sample was considered cytotoxic if the percentage of surviving cells was less than 60% of the negative control at one or more doses [18].

### 2.5. *Allium cepa*

Onions (*Allium cepa *L., 2*n* = 16) were obtained commercially and were placed in small jars with their basal ends dipped in distilled water. The newly sprouted roots (1-2 cm in length) were treated with organic extract of PM (5; 10; 15; 20 and 25 *μ*g/mL) for 24 h. DMSO was used as a negative control and benzo(a)pyrene (10 *μ*g/mL) as a positive control. The roots were exposed to treatment with organic extract and the controls for 24 h. Afterwards, the roots were removed from the bulbs and hydrolyzed with HCl 1N : acetic acid 45% (1 : 9) at 50°C for 5 min. The hydrolyzed root tips were squashed and stained with 2% aceto-orcein [24, 25]. Cytological abnormalities like micronuclei (MCN), bridges, breaks, and laggards were analyzed. The mitotic index (MI) was calculated for each treatment as the number of dividing cells per 1000 cells. 

## 3. Results and Discussion

### 3.1. Airborne Particulate Matter

Table 1 shows the air volume (m^3^), PM 2.5 *μ*m concentration (*μ*g/m^3^), and extractable organic matter (EOM) (*μ*g/m^3^) of the samples analyzed from the different sites.

The highest average PM 2.5 *μ*m values were detected at site 3 (94.54 to 132.73 *μ*g m^−3^), followed by site 2 (26.30; 45.14 *μ*g/m^3^) and site 1 (30.49 *μ*g/m^3^) (Table 1). The WHO has suggested that the maximum PM 2.5 concentration without incurring health risks is 25 *μ*g/m^3^ (24-hour mean) [26]. It has been demonstrated that for each 10 *μ*g/m^3^ increase in PM concentration, the risk of mortality from cardiopulmonary diseases increases 6%, while the risk of mortality from lung cancer rises 8% [27]. High levels of PM 2.5 were also detected in a previous study conducted from April to July 2010 at these same sites. The PM concentrations (site 3: 93 *μ*g/m^3^; site 2: 34–60 *μ*g/m^3^; site 1: 25 *μ*g/m^3^) reveal the persistence of this pollutant [15]. In both studies, the highest concentrations of PM 2.5 *μ*m were detected at site 3. A study performed in Wutong tunnel in Shenzhen city, China detected PM 2.5 concentrations of 110.8 *μ*g/m^3^ [28]. Both these results can be attributed to intense traffic and poor ventilation in tunnels. The implementation of road space rationing measures and improved ventilation could reduce the concentration of PM 2.5 in these tunnels.

In urban areas, high concentrations of PM derive mainly from motor vehicle emissions. In Rio de Janeiro city, vehicles account for 77% of emissions [29]. Despite improvements in the quality of fuels, high concentrations of PM are still detected in the city. This is due to the increased fleet of vehicles in the city, which over the last twenty years, has tripled from 838,521 to 2,529,432 [30]. Other urban areas with intense traffic also show high levels of PM 2.5: Beijing, China (66 *μ*g/m^3^) [31], Mexico City (47.20 *μ*g/m^3^) [32], Palermo, Italy (34.20 *μ*g/m^3^) [33], and Coimbatore, India (57.93 *μ*g/m^3^) [34]. The use of clean fuels and improved public transport are the best solutions for this problem, since they would result in lower PM emissions in urban areas.

### 3.2. Analysis of Polycyclic Aromatic Hydrocarbons (PAHs)

**Figure 1:**
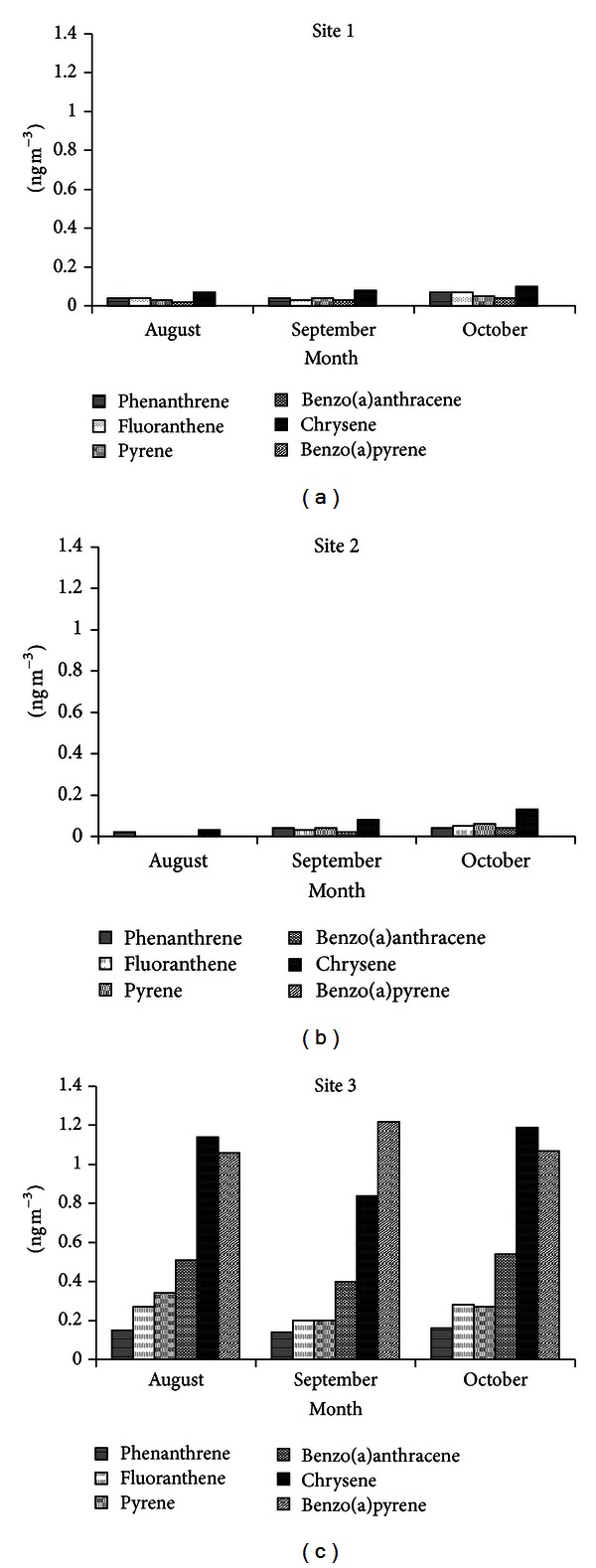
(a)–(c) PAH concentrations in ng/m^3^ of the three sites. Benzo(a)pyrene not detected at sites 1 and 2.

Figure 1 shows PAH concentrations in ng/m^3^ at the three sites.

In this study, the six PAHs evaluated are considered a priority in environmental monitoring [35]. PAHs are formed due to incomplete combustion [28]. A lot of effort has been invested in understanding their emissions, dynamic formation mechanisms, and atmospheric behavior. Exhausts from diesel fuel combustion and gasoline engines are a major source of PAH in the atmosphere [28, 36]. Urban traffic is the biggest contributor to PAH emissions in Rio de Janeiro city. Site 3 showed the highest concentrations of PAH; these results are in agreement with results obtained in July of the same year, for this same site [37]. These results too are in agreement with results obtained in other urban areas. In Europe, for example, the concentration of benzo(a)pyrene in high traffic areas varies from 1 to 5 ng/m^3^ [38]. In Sweden, in particular, the concentration of benzo(a)pyrene ranges from 1 to 2 ng/m^3^. These data have prompted political leaders in this country to stipulate a target concentration limit of 0.1 ng/m^3^ for benzo[a]pyrene [39]. In our results, benzo(a)pyrene was detected only in site 3 at a concentration of between 1.06 and 1.22 ng/m^3^. The fact that site 3 is enclosed and poorly ventilated may favor the accumulation of this PAH.

Sites 1 and 2 showed the lowest concentrations of the PAH studied. These results could be attributed to the fact that Rio de Janeiro is a coastal city, and the sea serves as an effective dispersant of pollutants [37, 40]. 

### 3.3. *Salmonella*/*Microsome* Assay

Table 2 shows the mutagenicity data for the organic extracts from airborne particulate matter in rev/m^3^. Cytotoxic effects were not detected for any of the samples analyzed. 

Mutagenic frameshift responses in the presence of metabolic activation were detected at sites 1 (August and September), 2 (August), and 3 (August, September, and October) (Table 2). These results can be attributed to the presence of promutagens such as PAH detected in this study. Highest mutagenic frameshift responses in the presence of metabolic activation were detected at site 3; this result may be related to high concentrations of PAH (Figure 1), resulting from intense emission by vehicles in this area of the city. Sites 1 (August) and 2 (August), winter season, showed higher values for rev/m^3^ than in April and May, autumn season, of the same year [15]. These results suggest that winter season, which is a dry season with low rainfall season, favors the accumulation of pollutants. Similar results were obtained in a study conducted in the city of Rio de Janeiro winter 1984 [41] and July of 2010 [37]. 

Mutagenic frameshift responses in the absence of metabolic activation were detected at sites 2 (August and September) and 3 (August, September, and October) (Table 2) which indicate the predominance of direct-acting frameshift activity in the airborne particulate material. The occurrence of direct frameshift mutagens adsorbed onto air particulate has been extensively demonstrated in urban samples collected throughout the world, and PAH derivatives, mainly nitro-PAH and oxygenated PAH, can be implicated [42–44]. These compounds may be derived from the emission of diesel. The highest mutagenic frameshift responses in the absence of metabolic activation values were detected at site 3. These results may be related to the emission of nitrocompounds at this site. Site 3 shows high flow of buses and trucks, contributing to the emission of compounds derived of diesel. Besides, the absence of pollutant dispersion factors may have contributed to the buildup of these pollutants.

The contribution of nitrocompounds to direct mutagenic activity was investigated through the *Salmonella/microsome* assay with specific strains YG1021 and YG1024. At the three assessment sites, the increase in mutagenic activity in overexpressing strains, YG1021 and YG1024 compared to the parental TA98, indicates the participation of nitroarenes and dinitroarenes in the total mutagenicity of the extracts studied. The presence of nitroarenes weas detected at site 1 (August, September, and October), 2 (August and October), and 3 (September). The presence of dinitroarenes was detected at site 1 (September and October), 2 (August and October), and 3 (September and October). It is well known that when nitrocompounds are implicated, the mutagenic response in the presence of S9 mix is decreased compared to the situations without S9 mix [8, 11]. This is because mammalian enzymes reduce nitroarenes all the way to arylamines, and arylamines are not direct-aging mutagens. The mutagenicity of these chemicals can be explained by their reoxidation by cytochrome P450-dependent enzymes to the arylhydroxylamines. However, it must be noted that generally arylamines exhibit a much lower mutagenicity in the presence of S9 mix than the corresponding nitroarenes in the absence of S9 mix [45].

These nitrocompounds result from direct emissions of diesel combustion and can be produced by atmospheric reactions of PAH with gaseous copollutants found in photochemical smog [44, 46]. In Brazil, the presence of nitrocompounds has also been observed in air samples from Rio de Janeiro [15, 37], Porto Alegre [17, 18, 47], and São Paulo [11, 44, 48]. 

### 3.4. *Allium cepa*

Tables 3, 4, and 5 show the mitotic index, percentage of mitosis stages, and mitotic aberrations in the root tip cells of *A. cepa* treated with organic extract derived from sites 1, 2, and 3, respectively.

The mitotic root meristems of* Allium cepa* have been used as cytogenetic materials for clastogenicity studies of physical and chemical agents since the early 1930s [49, 50]. In this work, we evaluated organic extracts from different sites of the city of Rio de Janeiro for cytotoxic/proliferation effect and chromosomal abnormalities. A reduced MI in relation to the negative control could indicate a cytotoxic effect, while a higher MI could be indicative of a proliferative effect [51]. In our results, we detected a cytotoxic effect in organic extracts at site 1 (August) (Table 3), site 2 (August) (Table 4), and site 3 (September to October) (Table 5). The positive control used in the test also presented a cytotoxic effect, as observed in the literature [52]. The reduction in the percentage of mitotic cells in certain stages observed at the three sites may indicate the need to disrupt the mitotic cycle in order to possibly repair the damage caused by the components of the organic extracts. 

Chromosome aberrations are characterized by changes in either chromosomal structure or the total number of chromosomes, which can occur both spontaneously and as a result of exposure to physical or chemical agents [14]. Structural chromosomal alterations may be induced by several factors, such as DNA breaks, inhibition of DNA synthesis, and replication of altered DNA [14, 53]. Two studies conducted in different regions of Yugoslavia to evaluate the occurrence of chromosomal abnormalities in the particulate matter deposited in the snow using the *Allium cepa* pointed to an increase in chromosomal abnormalities [54, 55]. Our results are in agreement with data from the literature. We detected significantly increased abnormalities, such as a bridge at site 2 (Table 4) and a micronucleus and bridge at site 3 (Table 5). A significant increase in breaks was observed at all three sites. Bridges noticed in the cells were probably formed by the breakage and fusion of chromosomes and chromatids [25]. Micronuclei are the result of damage in the parental cells, that is, either not repaired or wrongly repaired, and are easily observed in daughter cells as a similar structure to the main nucleus, but in a reduced size [14]. Micronucleus, bridge, and break abnormalities are attributed to clastogenic substances, which indicate that our results may indicate the clastogenic activity of the organic extracts. We call to attention that in some periods, the high concentrations of the extracts lead to a cytotoxic effect that can dissemble a clastogenic effect. 

According to [56], a complex mixture of hydrocarbons may present clastogenic and aneugenic activities or even induce cell death in *Allium cepa* genetic material. The authors also suggest that these actions mainly result from the presence of polycyclic aromatic hydrocarbons (PAHs) detected in the tested sample. Furthermore, *Allium cepa* assay has also the ability to detect nitrocompounds [25, 52]; therefore, these results may be related to the presence of these compounds in organic extracts.

The cytotoxic effect and the abnormalities detected in the present study can be attributed to the presence of PAH nitroderivatives in the organic extracts. As a response to the presence of pollutants, *Allium cepa* has a mechanism involving cytochrome P450- (CYP-) dependent mixed function oxidases with particular reference to ethoxyresorufin-*O*-deethylase (EROD) activity [57, 58]. According to this mechanism, ligands like PAH activate the hydrocarbon receptor (AhR), which in its inactive form resides in the cytoplasm in a complex with the molecular chaperones, hsp90 and p23 [59]. CYP1A1 activation may be a result of the weakening of the interactions between AhR and hsp90, thus releasing the AhR to translocate to the nucleus and activate the CYP1A1 gene [58]. This reinforces the ability of this plant species to detect this class of pollutants.

## 4. Conclusion

In conclusion, both PAH and nitroderivatives probably contributed to the detected airborne genotoxicity at different sites of Rio de Janeiro. The information generated in this study shows the importance of simple biological tests such as the *Salmonella/microsome* and *Allium cepa* to better characterize air pollution. Evaluation of the genotoxicity of urban airborne particulate matter performed by these tests is important as a basis for decision making by regulatory authorities.

## Figures and Tables

**Table 1:** Collection sites, air volume, PM 2.5 *µ*m concentration, and extractable organic matter (EOM) of the samples analyzed.

Site	Month	Air volume (m^3^) ± S.D.	PM 2.5 *µ*m individual values (*µ*g/m^3^)	PM 2.5 *µ*m (*µ*g/m^3^) ± S.D.	EOM (*µ*g/m^3^)
	August	1513 ± 36	17.51/18.63/13.63/45.09	23.71 ± 14.41	9.66
1	September	1582 ± 53	23.43/43.83/40.24/14.48	30.49 ± 13.89	7.92
	October	1623 ± 148	14.46/13.61/21.73/11.27	15.27 ± 4.51	5.54

	August	1541 ± 1	40.19/44.59/44.01/51.77	45.14 ± 4.83	7.76
2	September	1554 ± 31	13.76/34.66/41.58/15.20	26.30 ± 13.94	7.24
	October	1640 ± 194	22.48/36.47/11.74/25.08	23.94 ± 10.15	5.48

	August	430 ± 67	42.38/168.85/35.60/131.33	94.54 ± 66.00	20.93
3	September	420 ± 40	68.85/167.33/91.55/61.01	97.18 ± 48.52	21.44
	October	359 ± 6	77.15/239.77/75.68/138.33	132.73 ± 77.09	25.05

1: UERJ; 2: Avenida Brasil; 3: Rebouças tunnel; S.D.: standard deviation; airborne PM 2.5 *µ*m samples were collected for 24 h at sites 1 and 2 and 6 h at site 3. Six-h time filter saturation at site 3.

**Table 2:** Induced mutagenic by airborne particulate matter organic extracts (rev/m^3^).

Site	Month	TA98		YG1021		YG1024	
		−S9	+S9	−S9	+S9	−S9	+S9
	August	n.d.^a^	15.80 ± 1.40	13.90 ± 0.80	9.50 ± 1.40	38.20 ± 1.60	15.50 ± 1.40
1	September	n.d.^a^	13.00 ± 2.10	6.10 ± 1.30	n.d.^a^	15.80 ± 2.50	3.40 ± 0.80
	October	n.d.^a^	n.d.^a^	10.20 ± 1.30	6.40 ± 1.10	2.90 ± 0.60	n.d.^a^

	August	17.00 ± 1.90	1.50 ± 0.50	17.30 ± 1.80	5.70 ± 1.60	43.40 ± 8.50	10.70 ± 1.80
2	September	5.70 ± 0.90	n.d.^a^	13.90 ± 1.30	6.40 ± 1.24	11.70 ± 2.50	6.00 ± 0.70
	October	n.d.^a^	n.d.^a^	4.90 ± 0.70	n.d.^a^	3.80 ± 0.30	n.d.^a^

	August	39.60 ± 11.90	13.00 ± 2.30	4.40 ± 1.90	3.10 ± 0.80	28.30 ± 3.10	2.30 ± 0.80
3	September	56.40 ± 20.60	58.70 ± 11.80	63.00 ± 3.20	26.60 ± 3.90	57.00 ± 6.90	49.30 ± 3.90
	October	9.30 ± 1.80	46.50 ± 4.20	35.40 ± 0.80	18.30 ± 1.80	22.80 ± 2.00	6.00 ± 2.00

1: UERJ; 2: Avenida Brasil; 3: Rebouças tunnel. n.d.^a^: not detected. Negative control: DMSO for the mutagenicity assay without S9 mix was TA98, (28 ± 5); YG1021, (20 ± 2); YG1024, (18 ± 4). DMSO for the mutagenicity assay with S9 mix was TA98, (43 ± 3); YG1021, (35 ± 23); YG1024, (18 ± 5). Positive controls for the mutagenicity assay without S9 mix were 4-nitroquinoline oxide (0.5 *µ*g/plate) for TA98, (853 ± 72); YG1021, (719 ± 75); YG1024, (1021 ± 54). Positive controls for the mutagenicity assay with S9 mix were 2-aminofluorene (1 *µ*g/plate) for TA98, (214 ± 42); YG1021, (150 ± 32); YG1024, (219 ± 81).

**Table 3:** Mitotic index and percentage of mitosis stages and mitotic aberrations in the root tip cells of *A. cepa* treated with organic extract derived at site 1.

Month	*µ*g/mL	Mitotic index	% P.	% M.	% A.	% T.	% T.A.	% MCN	% Bridge	% Break	% Lagging
	NC	0.03 ± 0.00	1.40	0.90	0.50	1.10	0.07	0.05	0.00	0.00	0.02
	5	0.04 ± 0.01	3.20*	0.80	0.60	0.30	0.12	0.02	0.02	0.00	0.08
August	10	0.02 ± 0.00*	0.20*	1.00	0.40	1.00	0.35	0.20	0.10	0.00	0.05
	15	0.01 ± 0.01*	0.03*	0.30	0.10	0.20*	0.55	0.55	0.00	0.00	0.00
	20	0.01 ± 0.01*	0.02*	0.10*	0.03*	0.20*	0.05	0.03	0.00	0.02	0.00
	25	0.00 ± 0.00*	0.00*	0.00*	0.00*	0.00*	0.00	0.00	0.00	0.00	0.00
	NC	0.02 ± 0.01	0.80	0.60	0.20	0.50	0.15	0.00	0.05	0.05	0.05
	5	0.04 ± 0.02	1.10	1.30	0.80	0.50	0.54	0.04	0.20	0.08	0.22
September	10	0.07 ± 0.03	2.00	2.50*	1.50*	1.50	1.30*	0.10	0.40	0.40	0.40
	15	0.01 ± 0.01	0.50	0.30	0.30	1.30	0.60	0.40	0.05	0.05	0.10
	20	0.06 ± 0.05	2.00	2.00	1.00	1.40	1.08*	0.06	0.33	0.26	0.43
	25	0.01 ± 0.01	0.50	0.50	0.10	0.30	0.28	0.03	0.06	0.16	0.03
	NC	0.02 ± 0.00	1.40	0.40	0.00	0.00	0.10	0.08	0.00	0.02	0.00
	5	0.11 ± 0.05	7.00	0.70	0.40	2.60	0.10	0.00	0.00	0.07	0.03
October	10	0.05 ± 0.05	2.70	0.70	0.50	1.60	0.18	0.00	0.03	0.10	0.05
	15	0.06 ± 0.04	2.60	1.30	0.30	2.30	0.29*	0.07	0.05	0.12*	0.05
	20	0.05 ± 0.03	2.50	1.10	0.40	0.80	0.10	0.00	0.02	0.03	0.05
	25	0.06 ± 0.03	3.20	1.00	0.50	1.00	0.31	0.03	0.00	0.18	0.10

P.: prophase; M.: metaphase; A.: anaphase; T.: telophase; T.A.: total abnormalities; MCN.: micronucleus; NC.: negative control—DMSO; positive control = benzo(a)pyrene 10 *µ*g/mL (mitotic index = 0.00*; % T.A. = 0.20; % MCN = 0.16; % bridge = 0.04) *P ≤ 0.05.

**Table 4:** Mitotic index and percentage of mitosis stages and mitotic aberrations in the root tip cells of *A. cepa* treated with organic extract derived at site 2.

Month	*µ*g/mL	Mitotic index	% P.	% M.	% A.	% T.	% T.A.	% MCN	% Bridge	% Break	% Lagging
	NC	0.08 ± 0.03	4.50	1.90	0.40	1.10	0.30	0.18	0.07	0.00	0.05
	5	0.07 ± 0.03	3.20	1.60	0.90*	1.80	0.57	0.25	0.17	0.00	0.15
August	10	0.04 ± 0.01*	1.80*	0.80	0.40	0.60	0.41	0.03	0.06	0.16	0.16
	15	0.04 ± 0.01*	0.33*	3.26	0.56	0.36	0.75*	0.03	0.23*	0.26*	0.23
	20	0.04 ± 0.02*	2.53	1.23	0.46	0.76	0.19	0.00	0.00	0.16	0.03
	25	0.04 ± 0.01*	0.10*	1.40	0.90*	1.40	0.70	0.00	0.50*	0.10	0.10
	NC	0.02 ± 0.01	0.80	0.60	0.20	0.50	0.15	0.00	0.05	0.05	0.05
	5	0.04 ± 0.02	1.10	1.30	0.80	0.50	0.54	0.04	0.20	0.08	0.22
September	10	0.07 ± 0.03	2.00	2.50*	1.50*	1.50	1.30*	0.10	0.40	0.40	0.40
	15	0.01 ± 0.01	0.50	0.30	0.30	0.01*	0.60	0.40	0.05	0.05	0.10
	20	0.06 ± 0.05	2.00	2.00	1.00	1.40	1.08*	0.06	0.33	0.26	0.43
	25	0.01 ± 0.01	0.50	0.50	0.10	0.30	0.28	0.03	0.06	0.16	0.03
	NC	0.05 ± 0.02	3.90	0.50	0.20	0.30	0.30	0.10	0.00	0.10	0.10
	5	0.05 ± 0.01	2.00	1.50	0.70	1.30	0.67	0.00	0.17*	0.30	0.20
October	10	0.07 ± 0.01	1.70	1.80	0.80	3.20*	1.05	0.00	0.10	0.75	0.20
	15	0.00 ± 0.00	0.00	0.00	0.00	0.00	0.00	0.00	0.00	0.00	0.00
	20	0.00 ± 0.00	0.00	0.00	0.00	0.00	0.03	0.00	0.03	0.00	0.00
	25	0.00 ± 0.00	0.00	0.00	0.00	0.00	0.00	0.00	0.00	0.00	0.00

P.: prophase; M.: metaphase; A.: anaphase; T.: telophase; T.A.: total abnormalities; MCN.: micronucleus; NC.: negative control—DMSO; positive control = benzo(a)pyrene 10 *µ*g/mL (mitotic index = 0.00*; % T.A. = 0.20; % bridge = 0.10; % break =0.10) *P ≤ 0.05.

**Table 5:** Mitotic index and percentage of mitosis stages and mitotic aberrations in the root tip cells of *A. cepa* treated with organic extract derived at site 3.

Month	*µ*g/mL	Mitotic index	% P.	% M.	% A.	% T.	% T. A.	% MCN	% Bridge	% Break	% Lagging
	NC	0.04 ± 0.02	2.60	0.70	0.40	1.00	0.12	0.07	0.00	0.00	0.05
	5	0.03 ± 0.02	0.70	0.40	0.90	0.30	0.30	0.04	0.02	0.00	0.14
August	10	0.07 ± 0.03	2.70	2.20	0.90	2.10	0.50*	0.30	0.10	0.00	0.10
	15	0.06 ± 0.04	1.70	2.20	1.10*	1.00	0.26	0.10	0.13	0.00	0.03
	20	0.04 ± 0.02	1.60	1.40	0.70	1.00	0.16	0.10	0.03	0.00	0.03
	25	0.03 ± 0.01	1.30	0.90	0.60	0.60	0.15	0.00	0.00	0.00	0.15
	NC	0.08 ± 0.06	6.00	1.60	0.90	1.40	0.67	0.10	0.08	0.20	0.42
	5	0.04 ± 0.03	3.10	1.00	0.40	2.10	2.05*	1.63*	0.12	0.22	0.05
September	10	0.03 ± 0.03	1.00*	0.80	0.40	0.60	0.78	0.25	0.03	0.20	0.30
	15	0.10 ± 0.10	3.30	3.20	1.20	2.00	1.80	0.00	1.00*	0.60	0.20
	20	0.00 ± 0.00*	0.00*	0.00*	0.00	0.00	0.00	0.00	0.00	0.00	0.00
	25	0.02 ± 0.01*	1.00*	0.10*	0.00	0.70	0.12	0.00	0.00	0.10	0.02
	NC	0.08 ± 0.01	5.70	0.70	0.60	1.20	0.30	0.00	0.05	0.05	0.20
	5	0.09 ± 0.01	3.80	1.60	0.80	2.90	0.60	0.00	0.00	0.50*	0.10
October	10	0.06 ± 0.05	1.90*	0.70	0.40	2.70	0.28	0.10	0.03	0.05	0.10
	15	0.03 ± 0.01*	0.90*	0.70	0.50	0.80	0.40	0.20	0.10	0.00	0.10
	20	0.00 ± 0.00*	0.00*	0.00	0.00	0.00	0.00	0.00	0.00	0.00	0.00
	25	0.01 ± 0.02*	0.80*	0.00	0.03	0.03	0.33	0.00	0.03	0.00	0.00

P.: prophase; M.: metaphase; A.: anaphase; T.: telophase; T.A.: total abnormalities; MCN.: micronucleus; NC.: negative control—DMSO; positive control = benzo(a)pyrene 10 *µ*g/mL (mitotic index = 0.00*; % T.A. = 0.00; %) *P ≤ 0.05.
